# Low concordance between QIAreach QuantiFERON-TB, a novel interferon-gamma release assay, and QuantiFERON-TB Gold Plus, in a population-based survey in Blantyre, Malawi

**DOI:** 10.1128/jcm.01323-24

**Published:** 2024-12-19

**Authors:** Hannah M. Rickman, Mphatso D. Phiri, Hannah Mbale, Katherine C. Horton, Marc Y. R. Henrion, Emily S. Nightingale, James A. Seddon, Elizabeth L. Corbett, Peter MacPherson

**Affiliations:** 1 Clinical Research Department, London School of Hygiene & Tropical Medicine 4906 , London, United Kingdom; 2 Malawi Liverpool Wellcome Programme 560808 , Blantyre, Malawi; 3 Department of Clinical Sciences, Liverpool School of Tropical Medicine 608447 , Liverpool, United Kingdom; 4 Department of Infectious Disease Epidemiology, London School of Hygiene & Tropical Medicine 4906 , London, United Kingdom; 5 Desmond Tutu TB Centre, Stellenbosch University 26697 , Stellenbosch, South Africa; 6 Department of Infectious Disease, Imperial College London 4615 , London, United Kingdom; 7 School of Health & Wellbeing, University of Glasgow 3526 , Glasgow, United Kingdom; University of Manitoba, Winnipeg, Manitoba, Canada

**Keywords:** tuberculosis, Latent TB, IGRA, QIAreach, Mtb infection, *Mycobacterium tuberculosis*

## Abstract

Urgent improvements in the diagnosis and management of *Mycobacterium tuberculosis* infection are required to reach End TB goals. Conventional interferon-gamma release assays (IGRAs), such as QuantiFERON-TB Gold Plus (QFT-Plus), require substantial laboratory infrastructure and large blood volumes, limiting use in high-burden settings. The QIAreach QuantiFERON-TB (QIAreach QFT) was developed to overcome these challenges but has not previously been evaluated in field conditions in a low-income, high-burden country, or at scale in children. We performed a diagnostic evaluation of QIAreach QFT against QFT-Plus, in a cross-sectional IGRA survey in Blantyre, Malawi. We recruited a population-representative sample of children aged 1–4 years and adolescents and adults aged 10–40 years, from households and primary care. We calculated sensitivity, specificity, and Cohen’s kappa for QIAreach QFT against QFT-Plus, and constructed Bayesian hurdle-categorical models to compare quantitative test results. A total of 1,049 participants were recruited (64%: 1–4 years; 13%: 10–19 years; and 23%: 20–40 years). More participants had a positive QIAreach QFT result (32%) compared to QFT-Plus (15%). Over half of positive QIAreach QFT results had time-to-positivity of exactly 20 min, the assay cutoff. There was minimal agreement between QFT-Plus and QIAreach QFT results (*κ* = 0.26), which was lowest in children aged 1–4 years (*κ* = 0.13). Sensitivity and specificity of QIAreach QFT relative to QFT-Plus were 62% and 74%, respectively, with poor correlation between quantitative results. The suboptimal performance of QIAreach QFT, particularly in young children, suggests that it cannot currently be recommended for wider use and that the urgent need for an accessible test of Mtb infection remains unmet.

**IMPORTANCE:**

Almost a quarter of the world’s population has evidence of *Mycobacterium tuberculosis* (Mtb) infection. Monitoring and addressing this substantial burden of so-called “latent” tuberculosis (TB) infection will be critical to reach End TB targets. However, current interferon-gamma release assays (IGRAs) for Mtb infection are costly, and require a large volume of venous blood and significant laboratory processing, which are major barriers to their wider use in low-income countries. The novel QIAreach QuantiFERON-TB (QIAreach) assay has been designed as a more accessible alternative. We sought to evaluate it against a reference standard of QuantiFERON-TB Gold Plus, in a large cross-sectional survey in Blantyre, Malawi. To our knowledge, this is the first diagnostic evaluation of QIAreach QFT to be performed in a population-based survey in a low-income high-incidence setting, and to specifically focus on young children (a priority group for interventions targeting Mtb infection). In contrast to previous studies in other settings, we observed poor performance of QIAreach QFT, particularly in young children where there was little correlation between the novel test and the reference standard. This leads us to conclude that this test cannot be widely recommended for use in its current form; indeed manufacture is currently suspended. We believe our findings are of urgent importance to policymakers, clinicians, and researchers and underscore the importance of careful evaluation of new diagnostics in the contexts where they are intended to be used.

## INTRODUCTION

Tuberculosis (TB) kills more people than any other infectious disease and progress toward the World Health Organization (WHO) End TB elimination targets remains unacceptably slow. An estimated 23% of the world’s population have evidence of *Mycobacterium tuberculosis* (Mtb) infection (1), defined by persistent immunoreactivity to Mtb antigens—measured, for example, through a tuberculin skin test (TST) or interferon-gamma release assay (IGRA)—in the absence of clinical TB disease (2). Positive tests of Mtb infection are associated with a higher incidence of TB disease (3) and are therefore a tool for individual risk stratification and identification of those most likely to benefit from preventive treatment to reduce morbidity, mortality, and onward transmission. At population level, Mtb immunoreactivity provides valuable epidemiological information about TB transmission, and can be used to identify high-risk individuals, track trends over time, and measure the impact of interventions (4).

Existing tests for Mtb infection have substantial limitations. TSTs cross-react with *Bacillus Calmette–Guérin* (BCG) vaccination and environmental non-tuberculous mycobacteria and require two healthcare contacts for the test to be administered and then read. Newer, more specific skin tests such as C-Tb, Diaskintest, and C-TST retain the logistical burdens of TST, and evidence of their performance in young children and other priority population groups is limited (5). IGRAs measure T-cell responses to *Mtb-*specific antigens, but conventional IGRAs developed to date have required large-volume venous blood samples and considerable laboratory processing, creating barriers to use in low-resource, high-burden settings where they might be most impactful (2).

The QuantiFERON-TB Gold Plus (QFT-Plus) (Qiagen, Venlo, Netherlands), an established IGRA, requires 4 × 1 mL blood tubes: TB1 (which measures CD4-mediated responses to Mtb antigens), TB2 (which measures both CD4- and CD8-mediated responses to Mtb antigens), mitogen (positive control), and nil (negative control to adjust for background IFN-γ or heterophile antibodies), combining IFN-γ levels from these tubes to give a result (positive, negative or indeterminate, with the latter occurring due to low mitogen or high nil). By contrast, the novel QIAreach QuantiFERON-TB (QIAreach QFT) (Qiagen, Venlo, Netherlands; Conformité Européenne mark 2021), which was developed to overcome some of the operational challenges of conventional IGRAs (6), uses a single 1 mL blood sample (the equivalent of the QFT-Plus TB2 tube). Additionally, while QFT-Plus uses an enzyme-linked immunosorbent assay (ELISA) to measure IFN-γ production, QIAreach QFT uses a semi-automated lateral flow immunofluorescence device, embedded within an electronic “eStick” inserted into a battery-powered “eHub,” delivering results (positive or negative) within 20 min after overnight tube incubation, alongside a quantitative time-to-positivity.

Previously published evaluations of QIAreach QFT (Supplementary Materials 1) (7) include: a US study of 111 adults with TB risk factors (6); a study in Japan of adults with either active pulmonary TB (*n* = 41) or no TB risk factors (*n* = 42) (8); a 2022 study in Italy of 130 adults with microbiologically confirmed TB and 174 low-risk healthy volunteers (9); a 2023 Malaysian study of 178 participants aged 4–82 years screened for Mtb infection during TB contact investigation (10); a 2023 study in Vietnam of 261 adults with TB symptoms or risk factors recruited during community active case finding (11); and a 2024 study of 89 TB household contacts aged 6–90 in Chile (12). These studies report the sensitivity of QIAreach QFT relative to QFT-Plus ranging from 94% to 100%. Specificity ranged from 68% to 95%, and Cohen’s kappa from 0.53 to 0.98, with two more recent studies accounting for the lower specificity (72–68%) and kappa (0.56–0.53) results (11, 12). Three studies comparing quantitative QFT-Plus values to the QIAreach QFT time-to-positivity reported good correlation (6, 8, 10), with weaker correlation observed in one (12). Only one participant in the above studies was a child under 5, a key group at high risk of progression to active disease. The QIAreach QFT assay has not been evaluated in community-representative populations, nor under field conditions in a community setting in a low-income country. A 2022 WHO policy statement concluded that QIAreach QFT could not be adequately compared with WHO-recommended IGRAs (13), recommending further research. We, therefore, evaluated QIAreach QFT against QFT-Plus in a population-based IGRA survey in Blantyre, Malawi.

## MATERIALS AND METHODS

This diagnostic evaluation was embedded within the Timasamala (TB Immunoreactivity for Surveillance in Malawi) study: a cross-sectional survey investigating the population-level prevalence of *Mtb* immunoreactivity among young children, adolescents, and adults in Blantyre, Malawi. Methods are published in detail elsewhere (14). Blantyre is a densely populated, resource-limited urban setting with an estimated adult HIV prevalence of 14% (15), and an estimated adult prevalence of microbiologically confirmed TB of 150 per 100,000 (16).

### Study population and procedures

We aimed to recruit a community-representative sample of participants aged 1–4 years, and 10–40 years inclusive, from neighbourhoods of informal urban settlements in Blantyre, through recruitment in primary health clinics (PHCs) and households (14). Clinic-based recruitment of healthy children aged 1–4 years was performed in three PHCs in Blantyre, with eligible children identified from vaccination clinics, and from those accompanying others attending routine primary health services, such as maternity care, family planning, and cervical cancer screening. Participants were excluded if they were presenting to clinics because of their own symptomatic illness, or for HIV or TB care. In households, anyone in the relevant age groups and normally resident in the household was eligible for recruitment. Sample size was determined by the overall parent study (14).

Consenting participants and/or their guardians completed a questionnaire about demographics, household composition, socioeconomic status, medical history, TB exposure, self-/guardian-reported HIV status (and maternal HIV status for children), and current symptoms. Mid-upper arm circumference (MUAC) was measured for children aged 1–4 years. Children aged 1–4 years with a positive QFT-Plus were clinically reviewed; those with any TB symptoms were referred to local tertiary paediatric services for further investigation and management, while those without symptoms were referred for TB preventive treatment, in line with contemporaneous Malawi National TB Guidelines for TB contacts under the age of 5 (17).

### Laboratory procedures

All laboratory procedures were performed by trained technicians following the manufacturer’s instructions. A 5 mL venous blood sample was taken from all participants—1 mL directly into QIAreach QFT sample tubes and a further 4 mL into lithium heparin tubes for QFT-Plus. All samples were transferred to the laboratory at room temperature.

Within 10 h of collection, QIAreach QFT samples were incubated overnight (16–20 h) at 37°C, then centrifuged to separate plasma. This was mixed with QIAreach QFT plasma diluent buffer, pipetted into the sample port of eSticks, and processed with the QIAreach QFT eHub, with qualitative (positive/negative) and quantitative (time-to-positivity) results recorded.

For QFT-Plus, samples in lithium heparin tubes were transferred into QFT-Plus bottles in the laboratory and incubated at 37°C within 10 h of sample collection. After overnight (16–20 h) incubation, the samples were centrifuged to separate plasma, which was harvested and stored for up to 14 days at −20°C, to allow batch-processing. Manual IFN-γ ELISA was performed according to the manufacturer’s instructions. Results were analyzed using the QFT-Plus analysis software using the standard cutoff of 0.35 IU/mL. Technicians performing the QFT-Plus analysis were not aware of the QIAreach QFT results.

### Statistical analysis

All statistical analysis was performed using R version 4.2 (R Core Development Group, Vienna). Sensitivity, specificity, positive predictive value (PPV), and negative predictive value (NPV) of QIAreach QFT were calculated using the epiR package (18) against a reference standard of QFT-Plus. Calculations of Cohen’s kappa, PPV, and NPV were calculated excluding indeterminate QFT-Plus results. Cohen’s kappa was interpreted according to cutoffs from McHugh (19). The impact of covariates on specificity and sensitivity was evaluated using logistic regression. In a *post hoc* analysis, we also explored the effect of reclassifying QIAreach QFT results which were positive with a time-to-positivity of exactly 20 min (i.e., the assay cutoff) as negative, given the unexpectedly high number of such assays.

We evaluated the quantitative relationship between QIAreach QFT time-to-positivity and QFT-Plus TB2 IFN-γ levels among participants with a positive QIAreach QFT result (and excluding those with indeterminate QFT-Plus results). We modeled QIAreach QFT time-to-positivity on both a linear scale and on an inverse (1/TTP) rate scale, as we would expect this rate to be positively correlated with the concentration of IFN-y. We fitted locally estimated scatterplot smoothing curves to the data and, as there was evidence of a non-linear relationship, quantified correlation using Spearman’s rank coefficient. We constructed a Bayesian hurdle-ordered categorical regression model incorporating the effect of TB2 IFN-γ levels on (i) the probability of having a positive QIAreach QFT (hurdle) and (ii) the time-to-positivity for positive QIAreach QFT samples (Supplementary Materials 2). This was constructed in R using brms as an interface to Stan (20). We included the age category covariable, and an interaction term between TB2 and age category. We drew 4,000 samples for each parameter from the joint posterior distribution and summarized the percentage of TB2 results falling into each QIAreach QFT time-to-positivity interval using means and 95% uncertainty intervals. Priors were weakly informative, and models were checked by inspecting trace plots, posterior predictive plots, *R* statistics, and effective sample size measures.

## RESULTS

### Participants

In total, 1,049 participants had results for both QFT-Plus and QIAreach QFT (Table 1). Of these, 675 (64%) were aged 1–4 years, with 13% aged 10–19 years and 23% aged 20–40 years.

**TABLE 1:** Characteristics of participants^*a*^

Characteristic	Result for participants in age group (years)			
	1–4 (*n* = 675)	10–19 (*n* = 132)	20–40 (*n* = 242)	Overall (*n* = 1,049)
Age (years), median (IQR)	2.3 (1.50–3.44)	16.1 (12.9–18.6)	26.9 (23.0–32.4)	3.6 (1.92–19.0)
Female	329 (49%)	74 (56%)	165 (68%)	568 (54%)
Recruitment location				
Clinic	571 (85%)	0 (0%)	0 (0%)	571 (54%)
Community	104 (15%)	132 (100%)	242 (100%)	478 (46%)
HIV positive	4 (0.6%)	1 (0.8%)	25 (10%)	30 (3%)
HIV exposed uninfected	139 (21%)			
Current cough	132 (20%)	22 (17%)	13 (5%)	167 (16%)
Any current TB symptoms^*b*^	196 (29%)	28 (21%)	27 (11%)	251 (24%)
BCG vaccination	666 (99%)	Not asked	Not asked	
MUAC^*c*^	99 (15%)	Not measured	Not measured	
<12.5 cm	7 (1%)			
12.5 to <13.5 cm	60 (9%)			
≥13.5 cm	608 (90%)			
Previous TB treatment	1 (0.1%)	1 (0.8%)	2 (0.8%)	4 (0.4%)
QFT-Plus result				
Negative	524 (78%)	102 (77%)	171 (71%)	797 (76%)
Positive	71 (11%)	22 (17%)	63 (26%)	156 (15%)
Indeterminate	80 (12%)	8 (6%)	8 (3%)	96 (9%)
QIAreach QFT result				
Negative	505 (75%)	91 (69%)	121 (50%)	717 (68%)
Positive	170 (25%)	41 (31%)	121 (50%)	332 (32%)
Positive QIAreach TTP^*d*^				
20 min	117 (69%)	22 (54%)	36 (30%)	175 (53%)
<20 min	52 (31%)	19 (46%)	85 (70%)	156 (47%)

^*a*^ IQR: interquartile range. TB: tuberculosis. BCG: Bacille Calmette-Guérin. MUAC: mid-upper arm circumference. TTP: time-to-positivity. QIAreach: QIAreach QFT.

^*b*^ Any of fever, cough, weight loss, or night sweats (participants aged 10+), or fever, cough, night sweats, or weight loss/poor weight gain/failure to thrive (participants aged 1–4).

^*c*^ MUAC <12.5 cm may be used to indicate moderate malnutrition, while MUAC <13.5 cm may be used to designate those at risk of malnutrition (21).

^*d*^ Where QIAreach QFT is positive, the assay reports a time-to-positivity, up to a maximum of 20 min. We observed a high proportion of results with a reported time-to-positivity of exactly 20 min. Data missing for 1 participant.

Overall, 156 out of 1,049 (15%) QFT-Plus results were positive; more than two times as many participants (332 out of 1,049, 32%) had a positive QIAreach QFT. The prevalence of both QFT-Plus and QIAreach QFT positivity increased with age, with QIAreach-positivity higher than QFT-Plus in all age-groups. Overall, 96 out of 1,049 (9%) of QFT-Plus results were indeterminate, predominantly in young children (80 out of 675, 12%). The majority (88 out of 96, 92%) of indeterminate results occurred due to a low mitogen reading (Supplementary Materials 3).

### Agreement between QFT-Plus and QIAreach QFT

Minimal agreement was seen between QFT-Plus and QIAreach QFT results (Cohen’s *κ* = 0.26, 95% confidence interval [CI] = 0.20–0.32) (Table 2) (19). Agreement was lowest in children aged 1–4 years (0.13, 95% CI = 0.05–0.20) and highest (but still weak) in adults aged 20–40 years (0.40, 95% CI = 0.28–0.51).

**TABLE 2:** Test performance comparing QIAreach QFT with QFT-Plus^*d*^

Parameter	Result for group					
	Overall (*n* = 1,049)	Age group (years)			HIV status (age 20+)^*a*^	
		1–4 (*n* = 675)	10–19 (*n* = 132)	20–0 (*n* = 242)	Negative (*n* = 215)	Positive (*n* = 25)
QFT-Plus positive, QIAreach positive (true positives)	97	30	13	54	47	7
QFT-Plus positive, QIAreach negative (false negative)	59	41	9	9	8	1
QFT-Plus negative, QIAreach positive (false positives)	209	122	26	61	52	9
QFT-Plus negative, QIAreach negative (true negatives)	588	402	76	110	102	8
QFT-Plus indeterminate, QIAreach positive	26	18	2	6	6	0
QFT-Plus indeterminate, QIAreach negative	70	62	6	2	2	0
Sensitivity of QIAreach (95% CI)^*b*^	62% (54–70%)	42% (31–55%)	59% (36–79%)	86% (75–93%)	85% (73–94%)	88% (47–100%)
Specificity of QIAreach (95% CI)^*b*^	74% (71–77%)	77% (73–80%)	75% (65–83%)	64% (57–71%)	66% (58–74%)	47% (23–2%)
Positive predictive value of QIAreach (95% CI)^*c*^	32% (27–37%)	20% (14–27%)	33% (19–50%)	47% (38–56%)	47% (37–58%)	44% (20–70%)
Negative predictive value of QIAreach (95% CI)^*c*^	91% (88–93%)	91% (88–93%)	89% (81–95%)	92% (86–96%)	93% (86–97%)	89% (52–100%)
Cohen’s kappa^*b*^ (95% CI)	0.26 (0.20–0.32)	0.13 (0.05–0.20)	0.26 (0.09–0.42)	0.40 (0.28–0.51)	0.41 (0.29–0.53)	0.27 (−0.05 to 0.59)

^*a*^ Very few HIV-positive participants under 20 so data not shown here.

^*b*^ Sensitivity and specificity of QIAreach calculated against comparator QFT-Plus positivity or negativity.

^*c*^ Indeterminate QFT-Plus values excluded for the purposes of calculating predictive values and Cohen’s kappa.

^*d*^ CI: confidence interval. QIAreach: QIAreach QFT.

Against a reference standard of QFT-Plus, the overall sensitivity of QIAreach QFT was 62% (95% CI: 54–70%) with a specificity of 74% (95% CI: 71–77%). Sensitivity was markedly low (42%, 95% CI: 31–55%) in children aged 1–4 years, and increased significantly with age to 86% (95% CI: 75–93%) in adults aged 20–40 years (*P* < 0.001). Sensitivity was not significantly different in 25 adults with HIV (88%, 95% CI: 47–100%, *P* = 0.88). Specificity in children aged 1–4 years was 77% (95% CI: 73–80%), and decreased with age to 64% (95% CI: 57–71%) in adults aged 20–40 years (*P* = 0.002). Specificity was 47% (95% CI: 23–72%) in adults with HIV (*P* = 0.12).

In young children, neither sensitivity nor specificity was associated with maternal HIV status (HIV exposure) (*P* = 0.70 and *P* = 0.66, respectively) nor with MUAC status (*P* = 0.12 and *P* = 0.90, respectively) (Supplementary Materials 4).

### Possible sources of misclassification

As the QIAreach QFT uses only the equivalent of a TB2 tube, we sought to explore whether misclassifications had arisen from QFT-Plus samples which were only positive on TB1 and negative on TB2. Out of 156 positive QFT-Plus results, 30 (19%) had a positive TB1 only (Supplementary Materials 5). Of these, 18 (60%) returned a “false negative” QIAreach QFT result, representing 31% of the 59 “false negatives” (Table 2).

The QFT-Plus assay has a nil tube, which is subtracted from the TB1 and TB2 values to give a final adjusted reading, which is absent in QIAreach QFT. Out of 797 negative QFT-Plus results, 49 (6%) had an unadjusted TB1 or TB2 quantitative level higher than 0.46 IU/mL (equivalent to the test cutoff plus the median nil value), suggesting that an unadjusted TB2 level might give a false positive result (Supplementary Materials 6). Of these, 32 had a positive QIAreach QFT, representing 15% of the 209 “false positives” (Table 2).

### QIAreach QFT time-to-positivity

**Fig 1:**
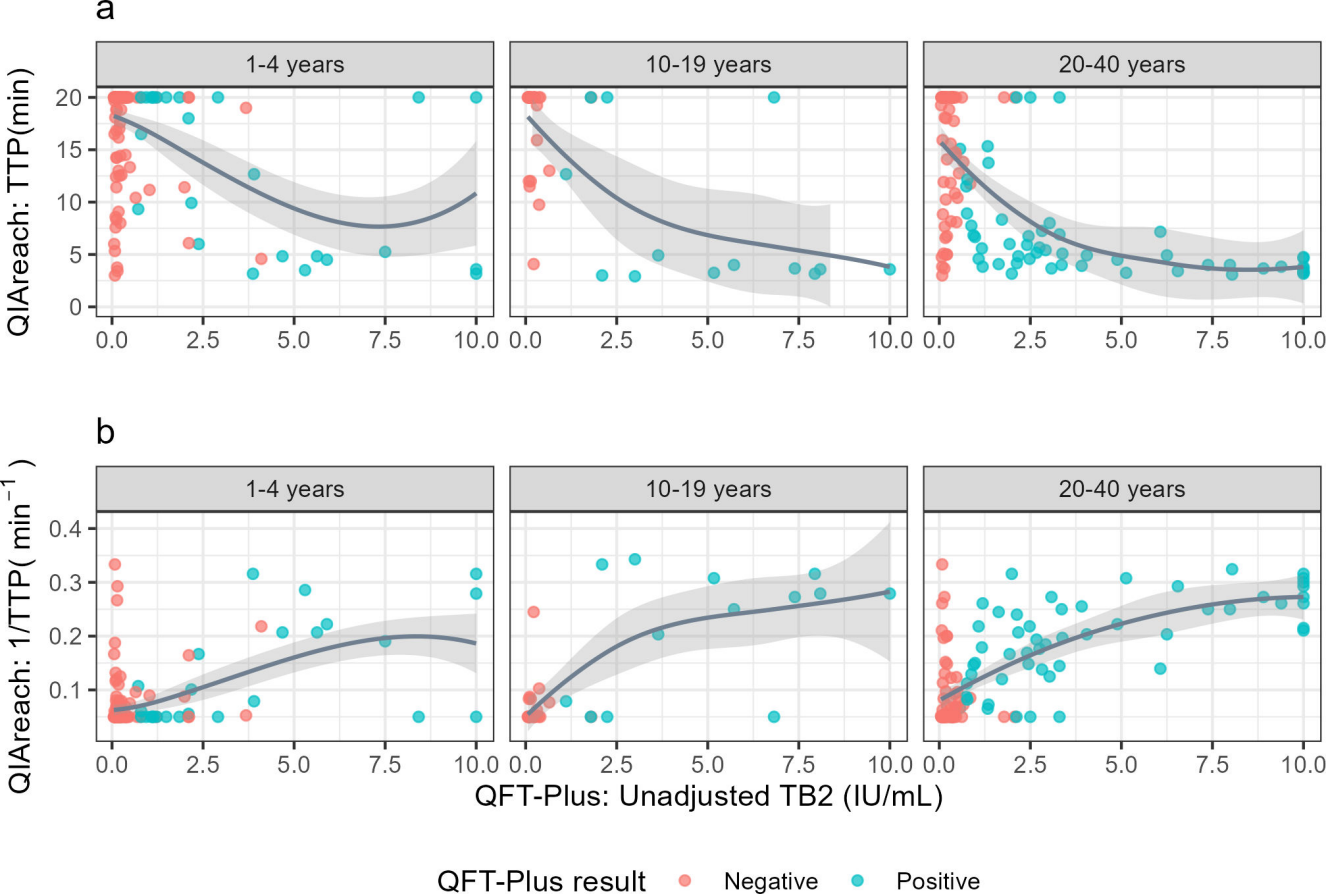
The relationship between QFT-Plus TB2 interferon-γ levels (not adjusted for nil values) and QIAreach QFT time-to-positivity (TTP) in participants with a positive QIAreach QFT, by age category. Locally estimated scatterplot smoothing (LOESS) functions were used to visualize the relationship, and are plotted in gray. In panel **a**, the raw QIAreach QFT TTP; in panel **b**, this is converted to a rate (1/TTP).

A higher QIAreach QFT time-to-positivity reflects a lower quantitative IFN-γ concentration. Overall, 53% of positive QIAreach QFT results had a time-to-positivity reported at exactly 20 min, the assay cutoff (Fig. 1). This was higher in children aged 1–4 years (69% of positive QIAreach QFT results) compared to adults aged 20–40 years (30% of positive results). About 15% of participants with a “borderline positive” QIAreach QFT result (positive at exactly 20 min) also had a positive QFT-Plus, compared to 50% of those with a QIAreach QFT time-to-positivity of less than 20 min; overall, 133 out of 208 (64%) of the “false positive” QIAreach QFT results (positive QIAreach QFT, negative QFT-Plus) had a time-to-positivity of exactly 20 min. Reclassifying these “borderline positive” results as negative resulted in an increased overall specificity of 90% (88–92%) but a reduced sensitivity of 47% (39–55%) (Supplementary Materials 7).

### Correlation between QIAreach QFT and QFT-Plus quantitative values

Among positive QIAreach QFT results, there was only moderate correlation between the QIAreach QFT time-to-positivity and quantitative TB2 levels (Fig. 1) (Spearman’s *ρ* = −0. 50). Correlation was weaker in young children (*ρ* = −0.26) compared to adolescents (*ρ* = −0.59) and adults (*ρ* = −0.59).

**Fig 2:**
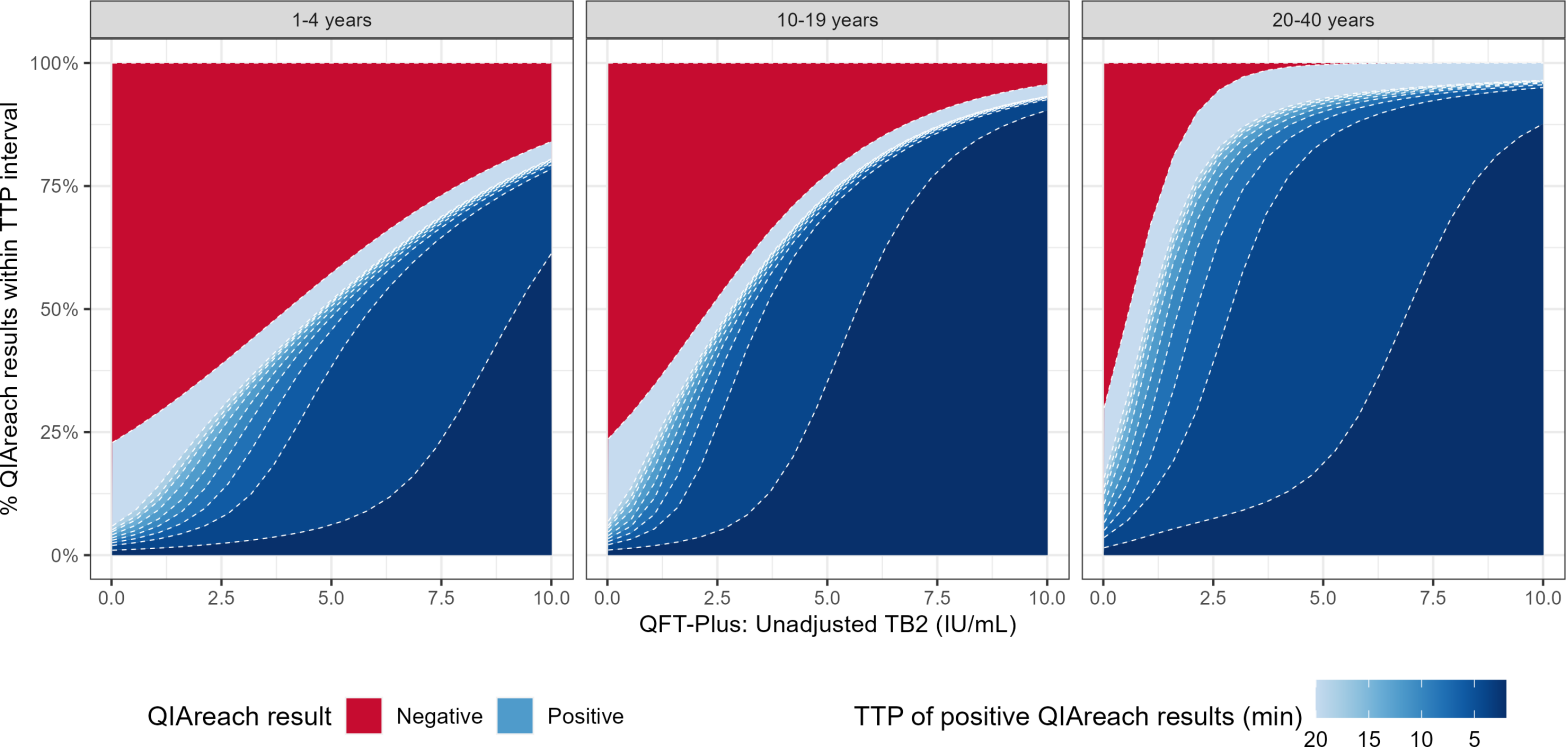
Posterior predictions from a hurdle categorical model of QIAreach QFT result and TTP at different levels of QFT-Plus TB2 IFN-γ, by age category. At each level of TB2, the *y*-axis designates the percentage of participants predicted to have negative (red) or positive (blue) QIAreach QFT result, with the intensity of blue indicating TTP, from the assay cutoff at exactly 20 mins (light blue) to lower TTP indicating more strongly positive results (dark blue). TTP, time to positivity.

We further explored the relationship between QFT-Plus TB2 concentration and QIAreach QFT results using a hurdle categorical model to account for non-linearity, and to incorporate the distributions of both binary positive/negative results, and the time-to-positivity of positive results (Fig. 2).

A low concentration of TB2 IFN-γ will (in the absence of an isolated high TB1) produce a negative QFT-Plus result. At a TB2 IGFN-γ concentration of 0.0 (zero) IU/mL, a similar proportion of young children (77%, 95% uncertainty interval [UI]: 74–81%), adolescents (76%, 95% UI: 68–84%) and adults (71%, 95% UI: 63–76%) were predicted to have a corresponding negative QIAreach QFT result. The remainder had a (likely falsely) positive QIAreach QFT result, with many (respectively: 16%, 95% UI: 14–20%; 17%, 95% UI: 11–24%; and 14%, 95% UI: 9–19%) positive only at the 20-min cutoff.

As TB2 levels increased, we observed a weaker increase in probability of both positive QIAreach QFT and of a lower time-to-positivity, among 1- to 4-year-olds than we did in older age groups. This reflects the lower sensitivity and correlation between quantitative results in younger children. At a TB2 level of 1.0 IU/mLl (which, in the absence of a high nil, would correspond to a positive QFT-Plus), the QIAreach QFT result was still predicted to be negative in 72% (95% UI: 67–75%) of 1- to 4-year-olds. At the same level of TB2, most (65%, 95% UI: 54–76%) adults aged 20–40 years had a positive QFT-Plus result. At an even higher TB2 of 5.0 IU/mL (likely to correspond to a strong positive QFT-Plus), 43% (95% UI: 26–59%) of QIAreach QFT results in 1- to 4-year-olds continued to be negative, while 99.7% (95% UI: 98–100%) of results in adults aged 20–40 were positive and 84% (95% UI: 74–91%) of adult participants had a time-to-positivity of less than 6 min.

## DISCUSSION

We observed poor concordance between QFT-Plus and QIAreach QFT in all age groups in this population-based survey in Blantyre, Malawi, in contrast to previous studies in other settings (6, 8–10). QIAreach QFT performance was notably poor in children aged 1–4 years, a priority group for interventions targeting Mtb infection. Our findings suggest that further optimization is needed before QIAreach QFT can be recommended for wider use, particularly in young children, and underscores the importance of careful evaluation of new diagnostics in the contexts where they are intended to be used.

Some discrepant results could be accounted for by factors inherent to the single-tube QIAreach QFT assay; 31% of QFT-Plus positive, QIAreach-negative samples (false negatives) were only positive on the QFT-Plus TB1 tube, while 15% of QFT-Plus negative, QIAreach-positive “false positives” could be accounted for by failure to adjust for the nil tube. However, this accounted for only a minority of discordance. There were twice as many positive QIAreach QFT as QFT Plus results, in line with the lower specificity reported in recent studies (11, 12), and half of the positive QIAreach results were “borderline,” with a time to positivity at the assay cutoff. Nevertheless, most of the QIAreach positive results were among QFT-Plus negative participants, driving the low sensitivity. Furthermore, we also noted poor quantitative correlation between TB2 IFN-γ concentrations and the QIAreach QFT time-to-positivity.

Several further factors could have contributed. First, this study was performed under real-world conditions in a low-resource setting. All protocols followed the standard manufacturer procedures and, when we first identified a high proportion of discordant results, a representative from the manufacturer visited our lab to review processing of both QFT-Plus and QIAreach QFT. No critical issues were identified. However, it is possible that sample collection and processing may have been less tightly controlled than in a central hospital, albeit representing realistic conditions for a community-based survey or a peripheral facility without an on-site laboratory. Variation in several pre-analytical factors, including time to incubation, duration of incubation, and intensity of tube shaking, are known to impact the results of conventional IGRAs (22, 23), and may be particularly relevant in the QIAreach QFT given the lack of control tubes.

Second, we cannot exclude a technical or manufacturing problem or batch variability between tubes or test reagents (24).

Third, the assay’s discriminatory performance may have been impacted by our cross-sectional population-based design. Most previous diagnostic evaluations have included participants with specific indications for Mtb infection testing (such as a discrete, defined TB contact or epidemiological risk factor) or microbiologically confirmed active TB, with or without “negative control” healthy volunteers in low-incidence settings (6, 8–10), which may lead to spectrum bias. By contrast, ours was a population-based study in a high-incidence setting, in which many people will have experienced some degree of TB exposure during their lifetimes. The population spectrum of immunological response to Mtb may have contributed to the high proportion of “borderline positive” QIAreach QFT results we observed, and to the poor concordance on a binary test.

Fourth, and most notably, we observed very different performance of QIAreach QFT for under 5-year-old children—who are a key target for diagnosis of Mtb infection due to their high risk of progression to active TB if infected. Sensitivity (42%), concordance (κ = 0.13), and correlation between quantitative results (*ρ* = −0.26) were very low in children aged 1–4 years, with predicted QIAreach QFT results at a given level of TB2 IFN-γ differing markedly from older ages. Previous evaluations of QIAreach QFT had only included one child under 5 years (6, 8–10). In the Malaysian study which included 22 children aged 4–17 years, all seven discordant results occurred in adults (10), while a study in Chile which included 20 children aged 6–17 reported a *κ* of 0.53 (12). A second Chilean study that compared QIAreach QFT to a novel IGRA and to TST observed poorer test agreement in participants aged 18–35 years, compared to those aged 36–65 years, which the authors speculate may relate to BCG effects (25). Young children in Malawi are highly exposed to a wide range of different pathogens and to BCG vaccination, and it is conceivable that non-specific immune activation or production of heterophile antibodies may have interfered with the performance of one or both IGRA assays (26). Of note, we also observed a higher proportion of indeterminate QFT-Plus results in young children, of which most were due to low mitogen. Our study population did also include 30 participants with HIV, more than previous evaluations, but this represented only 3% of all participants and we did not observe a significantly poorer test performance in PLHIV.

Notably, there is no true “gold standard” test for Mtb infection; agreement between different tests such as IGRA and TST is often modest (27), and both are known to have a poor predictive value for the development of subsequent TB (3). It was not possible to follow-up participants for development of TB disease (the ultimate outcome of interest): as incidence is low and often develops over months or years, this would have required a larger sample size and longer follow-up period than was feasible in this study; furthermore high-risk participants with positive QFT-Plus results were referred for TB preventive treatment, which would have further reduced this risk and caused confounding. However, in the absence of a fully developed test with high predictive performance for the development of active TB, WHO continues to recommend IGRAs including QFT-Plus as a suitable test for Mtb infection. QIAreach QFT has been proposed as being essentially equivalent to QFT-Plus, but this is not supported by our study findings.

The QIAreach QFT assay has clear benefits in terms of its acceptability to participants and ease of use in the laboratory (6). Conventional IGRAs are a routine part of clinical practice in many high-income settings, but their requirement for complex laboratory infrastructure is prohibitive for scale-up in the settings where they may be most impactful. Newer skin tests also offer promise but have significant limitations (5).

As global TB incidence shifts, understanding Mtb infection is critical both epidemiologically, and to direct interventions to prevent progression to disease, thereby reducing onward transmission and TB-associated morbidity and mortality. Implementation of TB preventive treatment remains unacceptably low, and tools for risk stratification of those most likely to benefit are lacking (2). This study suggests that further research is needed before QIAreach QFT can be recommended for wider use. Indeed manufacturing of the assay is currently suspended, meaning that the need for a portable, affordable, scalable IGRA remains unmet.

## Data Availability

Code and anonymized data required to replicate analyses are available at https://github.com/hannahrickman/qiareach.

## SUPPLEMENTAL MATERIAL

Supplemental material jcm.01323-24-s0001.docx Supplemental materials 1 to 7.

ASM does not own the copyrights to Supplemental Material that may be linked to, or accessed through, an article. The authors have granted ASM a non-exclusive, world-wide license to publish the Supplemental Material files. Please contact the corresponding author directly for reuse.

## References

[1] Houben R, Dodd PJ. 2016. The global burden of latent tuberculosis infection: a re-estimation using mathematical modelling. PLoS Med 13:e1002152. doi:10.1371/journal.pmed.1002152 27780211 PMC5079585

[2] World Health Organization. 2021. WHO consolidated guidelines on tuberculosis. Module 1: prevention. Tuberculosis preventive treatment. 39298638

[3] Hamada Y, Gupta RK, Quartagno M, Izzard A, Acuna-Villaorduna C, Altet N, Diel R, Dominguez J, Floyd S, Gupta A, et al.. 2023. Predictive performance of interferon-gamma release assays and the tuberculin skin test for incident tuberculosis: an individual participant data meta-analysis. E Clin Med 56:101815. doi:10.1016/j.eclinm.2022.101815 PMC9829704 36636295

[4] Rickman HM, Kamchedzera W, Schwalb A, Phiri MD, Ruhwald M, Shanaube K, Dodd PJ, Houben RMGJ, Corbett EL, MacPherson P. 2022. Know your tuberculosis epidemic–Is it time to add Mycobacterium tuberculosis immunoreactivity back into global surveillance? PLOS Glob Public Health 2:e0001208. doi:10.1371/journal.pgph.0001208 36962621 PMC10021854

[5] Krutikov M, Faust L, Nikolayevskyy V, Hamada Y, Gupta RK, Cirillo D, Mateelli A, Korobitsyn A, Denkinger CM, Rangaka MX. 2022. The diagnostic performance of novel skin-based in-vivo tests for tuberculosis infection compared with purified protein derivative tuberculin skin tests and blood-based in vitro interferon-γ release assays: a systematic review and meta-analysis. Lancet Infect Dis 22:250–264. doi:10.1016/S1473-3099(21)00261-9 34606768

[6] Stieber F, Howard J, Manissero D, Boyle J, Ndunda N, Love J, Yang M, Schumacher A, Uchiyama R, Parsons S, Miller C, Douwes H, Mielens Z, Laing T, Nikolayevskyy V. 2021. Evaluation of a lateral-flow nanoparticle fluorescence assay for TB infection diagnosis. Int J Tuberc Lung Dis 25:917–922. doi:10.5588/ijtld.21.0391 34686234 PMC8544925

[7] Ortiz-Brizuela E, Apriani L, Mukherjee T, Lachapelle-Chisholm S, Miedy M, Lan Z, Korobitsyn A, Ismail N, Menzies D. 2023. Assessing the diagnostic performance of new commercial interferon-γ release assays for Mycobacterium tuberculosis infection: a systematic review and meta-analysis. Clin Infect Dis 76:1989–1999. doi:10.1093/cid/ciad030 36688489 PMC10249994

[8] Fukushima K, Akagi K, Kondo A, Kubo T, Sakamoto N, Mukae H. 2022. First clinical evaluation of the QIAreach^TM^ QuantiFERON-TB for *Mycobacterium tuberculosis* infection and active pulmonary disease. Pulmonol 28:6–12. doi:10.1016/j.pulmoe.2021.07.003 34362702

[9] Saluzzo F, Mantegani P, Poletti de Chaurand V, Cirillo DM. 2022. QIAreach QuantiFERON-TB for the diagnosis of Mycobacterium tuberculosis infection. Eur Respir J 59:6–9. doi:10.1183/13993003.02563-2021 PMC8943267 34675051

[10] Aziz ZA, Noordin NM, Wan Mohd WM, Kasim MA. 2023. First evaluation of the performance of portable IGRA, QIAreach® QuantiFERON®-TB in intermediate TB incidence setting. PLoS One 18:e0279882. doi:10.1371/journal.pone.0279882 36763619 PMC9916628

[11] Vo LNQ, Tran TTP, Pham HQ, Nguyen HT, Doan HT, Truong HT, Nguyen HB, Nguyen HV, Pham HT, Dong TTT, Codlin A, Forse R, Mac TH, Nguyen NV. 2023. Comparative performance evaluation of QIAreach QuantiFERON-TB and tuberculin skin test for diagnosis of tuberculosis infection in Viet Nam. Sci Rep 13:15209. doi:10.1038/s41598-023-42515-1 37709844 PMC10502094

[12] Ruiz-Tagle C, García P, Hernández M, Balcells ME. 2024. Evaluation of concordance of new QuantiFERON-TB gold plus platforms for Mycobacterium tuberculosis infection diagnosis in a prospective cohort of household contacts. Microbiol Spectr 12:e0046924. doi:10.1128/spectrum.00469-24 38975791 PMC11302262

[13] World Health Organization. 2022. Use of alternative interferon- gamma release assays for the diagnosis of TB infection: WHO policy statement.

[14] Rickman HM, Phiri MD, Feasey HRA, Mbale H, Nliwasa M, Semphere R, Chagaluka G, Fielding K, Mwandumba HC, Horton KC, Nightingale ES, Henrion MYR, Mbendera K, Mpunga JA, Corbett EL, MacPherson P. 2024. Tuberculosis Immunoreactivity Surveillance in Malawi (Timasamala)-A protocol for a cross-sectional Mycobacterium tuberculosis immunoreactivity survey in Blantyre, Malawi. PLoS One 19:e0291215. doi:10.1371/journal.pone.0291215 38787869 PMC11125513

[15] Malawi Ministry of Health. 2022. Malawi population-based HIV impact assessment (MPHIA) 2020-2021: final report. Vol. 2.

[16] Feasey HRA, Khundi M, Nzawa Soko R, Nightingale E, Burke RM, Henrion MYR, Phiri MD, Burchett HE, Chiume L, Nliwasa M, Twabi HH, Mpunga JA, MacPherson P, Corbett EL. 2023. Prevalence of bacteriologically-confirmed pulmonary tuberculosis in urban Blantyre, Malawi 2019-20: Substantial decline compared to 2013-14 national survey. PLOS Glob Public Health 3:e0001911. doi:10.1371/journal.pgph.0001911 37862284 PMC10588852

[17] Malawi Ministry of Health. 2018. Malawi National Tuberculosis & Leprosy Guidelines.

[18] Stevenson M, epiR S. 2023. Tools for the analysis of epidemiological data. R package version 2.0.66.

[19] McHugh ML. 2012. Lessons in biostatistics interrater reliability: the kappa statistic. Biochem Med 22:276–282. doi:10.11613/BM.2012.031 PMC3900052 23092060

[20] Bürkner PC. 2017. Brms: An R package for Bayesian multilevel models using Stan. J Stat Softw 80. doi:10.18637/jss.v080.i01

[21] World Health Organization. 2013 Guideline: updates on the management of severe acute malnutrition in infants and children. 24649519

[22] Shanaube K, De Haas P, Schaap A, Moyo M, Kosloff B, Devendra A, Raby E, Godfrey-Faussett P, Ayles H. 2010. Intra-assay reliability and robustness of QuantiFERON(R)-TB Gold In-Tube test in Zambia. Int J Tuberc Lung Dis 14:828–833. 20550764

[23] Gaur RL, Pai M, Banaei N. 2013. Impact of blood volume, tube shaking, and incubation time on reproducibility of QuantiFERON-TB gold in-tube assay modified title: impact of blood volume, tube shaking, and incubation time on reproducibility of QuantiFERON-TB gold in-tube assay. J Clin Microbiol 51:3521–3526. doi:10.1128/JCM.01627-13 23966505 PMC3889728

[24] Slater M, Parsonnet J, Banaei N. 2012. Investigation of false-positive results given by the QuantiFERON-TB gold in-tube assay. J Clin Microbiol 50:3105–3107. doi:10.1128/JCM.00730-12 22785197 PMC3421800

[25] Saint-Pierre G, Conei D, Cantillana P, Raijmakers M, Vera A, Gutiérrez D, Kennedy C, Peralta P, Ramonda P. 2023. Comparison of two tuberculosis infection Tests in a South American tertiary hospital: STANDARD F TB-Feron FIA vs. QIAreachTM QuantiFERON-TB. Diagnostics (Basel) 13:1162. doi:10.3390/diagnostics13061162 36980470 PMC10046924

[26] Zhou G, Luo Q, Luo S, Chen H, Cai S, Guo X, He J, Xia Y, Li H, Zhou Y, Zhang Y, Song C. 2023. Indeterminate results of interferon gamma release assays in the screening of latent tuberculosis infection: a systematic review and meta-analysis. Front Immunol 14:1170579. doi:10.3389/fimmu.2023.1170579 37256138 PMC10225525

[27] Venkatappa TK, Punnoose R, Katz DJ, Higgins MP, Banaei N, Graviss EA, Belknap RW, Ho CS. 2019. Comparing QuantiFERON-TB gold plus with other tests to diagnose Mycobacterium tuberculosis infection. J Clin Microbiol 57:1–9. doi:10.1128/JCM.00985-19 PMC6813010 31462550

