# Supplementary material for: Low concordance between QIAreach QuantiFERON-TB, a novel interferon-gamma release assay, and QuantiFERON-TB Gold Plus, in a population-based survey in Blantyre, Malawi
Source: J Clin Microbiol. 2024 Dec 19;63(1):e01323-24. doi: 10.1128/jcm.01323-24 (PMC11784431; doi:10.1128/jcm.01323-24)
Supplement: Supplemental material — s 1 to 7. [file jcm.01323-24-s0001.docx]

# Supplementary materials

1. Results of previous comparative studies of QIAreach QFT vs other tests of Mtb infection
2. Model specification for Bayesian hurdle ordinal categorical model
3. Analysis of indeterminate QFT-Plus results
4. Sensitivity and specificity of QIAreach QFT vs QFT-Plus by nutritional and HIV-exposure status in young children
5. QFT-Plus results positive on only TB1 tube
6. QFT-Plus results negative only when nil-adjusted
7. Sensitivity analysis reclassifying QIAreach QFT with time-to-positivity of 20 minutes as negative

| **Study** | **Population** | **2x2 table** | **Sens** | **Spec** | **Agreement** | **Quantitative** |
| --- | --- | --- | --- | --- | --- | --- |
| Stieber et al, 2021  IJTLD | USA  Aged 18+  111 participants (207 samples) with and without various risk factors for TB  -current or previous TB  -known positive TST or IGRA  -HIV (9 participants)  -TB contact/epi risk factors | Comparator: QFT-Plus   \|  \| QIA+ \| QIA- \| \| --- \| --- \| --- \| \| QFT + \| 68 \| 0 \| \| QFT - \| 6 \| 129 \| \| Ind \| 3 \| 0 \| | 100% vs QFT-Plus | 96% vs QFT-Plus | OPA: 97%  κ: 0.94 | Not formally analysed but good correlation in graph |
| Fukushima et al, 2021  Pulmonology | Japan  Aged 20+  1. Microbiologically-confirmed active TB (n=41; median age 82; 7 immunosuppressed)  2. Healthy/low risk (n=42) | Comparator: QFT-Plus   \|  \| QIA+ \| QIA- \| \| --- \| --- \| --- \| \| QFT + \| 41 \| 0 \| \| QFT - \| 1 \| 41 \| | 100% vs QFT-Plus  100% vs active TB | 98% vs QFT-Plus  98% vs healthy controls | OPA: 99%  κ: 0.98 | R -0.913 and -0.918 for log-transformed TTP vs IFN-γ |
| Saluzzo et al, 2022  Eur Resp J | Italy  Adults  1. Microbiologically-confirmed TB (treated and untreated) (n=130)  2. Low-risk healthy volunteers (n=174) | Comparator: TB disease, healthy controls, QFT-Plus  *Overall individual numbers not available* | 94-95% vs active TB  99% vs QFT-Plus | 98% vs healthy volunteers  93% vs QFT-Plus | OPA: 96%  κ: 0.96 | Not analysed |
| Aziz et al, 2023  PLOS ONE | Malaysia  TB contacts aged 4-82 years (n=178)  (median age 15) | Comparator: QFT-Plus   \|  \| QIA+ \| QIA- \| \| --- \| --- \| --- \| \| QFT + \| 55 \| 2 \| \| QFT - \| 7 \| 114 \| | 96% vs QFT-Plus | 94% vs QFT-Plus | OPA: 95%  κ: 0.88 | R -0.7984 and -0.7961 for untransformed IFN-γ vs TTP |
| Saint-Pierre et al, 2023  Diagnostics | Chile  Aged 18+ (n=76)  1. Indications for TB infection testing (TB contact, HIV, due to receive immunosuppressive drugs)  2. Low risk and previously TST negative  *(unclear if included in these results)* | Comparator: TST   \|  \| QIA+ \| QIA- \| \| --- \| --- \| --- \| \| TST + \| 30 \| 6 \| \| TST - \| 15 \| 25 \| | 83% vs TST | 63% vs TST | OPA: 72%  κ: 0.45  *Under 36:* OPA: 62% , κ: 0.26  *Over 36:* OPA: 81%, κ: 0.61 | Not analysed |
| Vo et al, 2023  Nature Scientific Reports | Vietnam  Aged 18+ (median age 61), n=261  Recruited at community active case finding activities and meeting at least one of the following criteria: TB exposure, HIV, diabetes, homelessness, illicit drug use, limited access to healthcare, TB symptoms | Comparator: QFT-Plus   \|  \| QIA+ \| QIA- \| \| --- \| --- \| --- \| \| QFT + \| 65 \| 1 \| \| QFT - \| 54 \| 141 \|   Comparator: TST at 10mm   \|  \| QIA+ \| QIA- \| \| --- \| --- \| --- \| \| TST + \| 16 \| 7 \| \| TST - \| 68 \| 109 \| | 98% vs QFT-Plus  70% vs TST (10mm) | 72% vs QFT-Plus  62% vs TST (10mm) | *Vs QFT-Plus*  OPA: 79%  κ: 0.56  *Vs TST (10mm)*  OPA: 63%  κ: 0.14 | Corrected mean IFN-γ in QFT-Plus positive participants higher in QFT-Plus positive vs QIAreach-positive participants (1.78 vs 1.0 IU/mL) |
| Ruiz-Tagle, 2024  Microbiology Spectrum | Chile  Aged 6-90 (median age 29), including 20 children between 6-17 years, n=89  All TB household contacts | Comparator: QFT-Plus   \|  \| QIA+ \| QIA- \| \| --- \| --- \| --- \| \| QFT + \| 23 \| 0 \| \| QFT - \| 21 \| 45 \| | 100% vs QFT-Plus | 68% vs QFT-Plus | OPA: 76%  κ: 0.53 | Spearman’s correlation: -0.81 for TB2 IFN-γ vs QIAreach TTP |

Supplementary Materials 1 - Results of previous comparative studies of QIAreach QFT vs other tests of Mtb infection. QFT: Quantiferon-Plus. QIA: QIAreach QFT. OPA: Overall percentage agreement. κ:Cohen’s kappa. TTP: time-to-positivity. TST: tuberculin skin test. IGRA: interferon-gamma release assay. IFN-γ: interferon gamma

Supplementary Materials 2 - Model specification for Bayesian hurdle ordinal categorical model

This model consists of two components.

i) Hurdle component

This models the probability for each individual $\pi_{i}$ that the QIAreach QFT result $Yi$ is negative, or equivalently that the time-to-positivity ${TTP}_{i}$ is greater than the assay cut-off of 20 minutes, at a given level of QFT-Plus TB2 ${TB2}_{i}$, and age category ${age}_{i}$.

$\pi_{i}=\Pr\left( Y_{i}=0 \right)= Pr({TTP}_{i} > 20)$

$\mathrm{logit}\left( \pi_{i} \right)= \alpha_{0,hu} + \alpha_{1,hu}\cdot{TB2}_{i} + \alpha_{2,hu}\cdot{age}_{i} + \alpha_{3,hu}\cdot{TB2}_{i}\cdot{age}_{i}$

ii) Cumulative component

For positive QIAreach results (${TTP}_{i} \leq20$), the cumulative component models the cumulative probabilities $\theta_{ik}$ that the time to positivity ${TTP}_{i}$ is in or below a specific time-to-positivity bracket $k$, at a given level of QFT-Plus TB2 ${TB2}_{i}$, and age category ${age}_{i}$.

Time-to-positivity was categorised into 2-minute brackets for the purposes of visualisation and interpretability, and because a significant proportion of positive results had a time-to-positivity of exactly 20 minutes, the assay cut-off, which was felt to be an important separate category.

$\theta_{ik}=Pr({TTP}_{i} \leq k)$

${\mathrm{cauchit}(\theta}_{ik})= \beta_{0} + \beta_{1}\cdot{TB2}_{i} + \beta_{2}\cdot{age}_{i} + \beta_{3}\cdot{TB2}_{i}\cdot{age}_{i}$

The code to reproduce the model is available in our online repository.

Supplementary Materials 3 - Analysis of indeterminate QFT-Plus results

1. Reason for indeterminate values

| Reason for indeterminate QFT-Plus | Age group (years) | | | |
| --- | --- | --- | --- | --- |
|  | 1-4 | 10-19 | 20-40 | Overall |
| High nil | 3 (4%) | 1 (13%) | 4 (50%) | 8 (8%) |
| Low mitogen | 77 (96%) | 7 (88%) | 4 (50%) | 88 (92%) |

1. QIAreach results for those with indeterminate values due to high nil or low mitogen

|  | Age group (years) | | | |
| --- | --- | --- | --- | --- |
|  | 1-4 | 10-19 | 20-40 | Overall |
| QFT-Plus indeterminate due to high nil | | | | |
| QIAreach negative | 2 (67%) | 1 (100%) | 1 (33%) | 4 (50%) |
| QIAreach positive | 1 (33%) | 0 (0%) | 3 (67%) | 4 (50%) |
| QFT-Plus indeterminate due to low mitogen | | | | |
| QIAreach negative | 60 (78%) | 5 (71%) | 1 (25%) | 66 (75%) |
| QIAreach positive | 17 (22%) | 2 (29%) | 3 (75%) | 22 (25%) |

1. MUAC results and HIV exposure for young children (aged 1-4) with indeterminate QFT-Plus results

| QFT-Plus result | MUAC (cm) | | | HIV exposure status | |
| --- | --- | --- | --- | --- | --- |
|  | <12.5 | 12.5 - <13.5 | ≥13.5 | HIV exposed | HIV unexposed |
| Indeterminate – high nil | 0 (0%) | 0 (0%) | 3 (100%) | 0 (0%) | 3 (100%) |
| Indeterminate – low mitogen | 2 (3%) | 9 (12%) | 66 (86%) | 13 (17%) | 64 (83%) |
| Participants without indeterminate results | 5 (1%) | 51 (9%) | 539 (91%) | 130 (22%) | 1. (78%) |

MUAC: Mid-upper arm circumference. CI: Confidence interval. QIAreach: QIAreach QFT.

Supplementary Materials 4 - Sensitivity and specificity of QIAreach QFT vs QFT-Plus by nutritional and HIV-exposure status in young children

|  | **All aged 1-4 years** | **MUAC normal (>13.5cm)** | **MUAC low (≤13.5cm)** | **HIV Unexposed** | **HIV Exposed** |
| --- | --- | --- | --- | --- | --- |
|  | **(n = 675)** | **(n = 576)** | **(n = 99)** | **(n = 532)** | **(n =143)** |
| QFT-Plus Positive, QIAreach Positive ("true positives") | 30 | 21 | 9 | 23 | 7 |
| QFT-Plus Positive, QIAreach Negative ("false negative") | 41 | 35 | 6 | 33 | 8 |
| QFT-Plus Negative, QIAreach Positive ("false positives") | 122 | 106 | 16 | 97 | 25 |
| QFT-Plus Negative, QIAreach Negative ("true negatives") | 402 | 351 | 51 | 312 | 90 |
| QFT-Plus Indeterminate, QIAreach Positive | 18 | 13 | 5 | 16 | 2 |
| QFT-Plus Indeterminate, QIAreach Negative | 62 | 50 | 12 | 51 | 11 |
| Sensitivity of QIAreach  (95% CI) ^1^ | 42%  (31%-55%) | 38%  (25%-51%)  - | 60%  (32%-84%)  *p=0.124* | 41%  (28%-55%)  - | 47%  (21%-73%)  *p=0.697* |
| Specificity of QIAreach  (95% CI) ^1^ | 77%  (73%-80%) | 77%  (73%-81%)  - | 76%  (64%-86%)  *p=0.901* | 76%  (72%-80%)  - | 78%  (70%-85%)  *p=0.658* |
| Positive predictive value of QIAreach (95% CI) ^2^ | 20%  (14%-27%) | 17%  (11%-24%) | 36%  (18%-57%) | 19%  (13%-27%) | 22%  (9%-40%) |
| Negative predictive value of QIAreach (95% CI) ^2^ | 91%  (88%-93%) | 91%  (88%-94%) | 89%  (78%-96%) | 90%  (87%-93%) | 92%  (85%-96%) |
| Cohen’s kappa^2^  (95% CI) | 0.13  (0.05-0.20) | 0.09  (0.01-0.17) | 0.29  (0.08-0.49) | 0.12  (0.03-0.20) | 0.17  (0.01-0.32) |

^1^Sensitivity and specificity of QIAreach calculated against comparator QFT-Plus positivity or negativity. ^2^Indeterminate values excluded for the purposes of calculating predictive values and Cohen’s kappa.

MUAC: mid-upper arm circumference. CI: Confidence interval. QIAreach: QIAreach QFT

Supplementary Materials 5 - QFT-Plus results positive on only TB1 tube

|  | Age group (years) | | | |
| --- | --- | --- | --- | --- |
| QFT-Plus positive results | 1-4 | 10-19 | 20-40 | Overall |
| TB1 & TB2 positive | 22 (31.0%) | 15 (68.2%) | 50 (79.4%) | 87 (55.8%) |
| TB2 only positive | 29 (40.8%) | 5 (22.7%) | 5 (7.9%) | 39 (25.0%) |
| ***TB1 only positive*** | 20 (28.2%) | 2 (9.1%) | 8 (12.7%) | 30 (19.2%) |
| ***QIAreach results amongst QFT-Plus results positive only on TB1*** | | | | |
| ***QIAreach negative*** | 13 (65.0%) | 2 (100.0%) | 3 (37.5%) | 18 (60.0%) |
| ***QIAreach positive*** | 7 (35.0%) | 0 (0.0%) | 5 (62.5%) | 12 (40.0%) |

Supplementary Materials 6 - QFT-Plus results negative only when nil-adjusted

|  | Age group (years) | | | |
| --- | --- | --- | --- | --- |
| QFT-Plus negative results | 1-4 | 10-19 | 20-40 | Overall |
| QFT-Plus negative results which are negative only when nil-adjusted | 29 (5.5%) | 4 (3.9%) | 16 (9.4%) | 49 (6.1%) |
| ***QIAreach results amongst QFT-Plus results negative only when nil-adjusted*** | | | | |
| ***QIAreach negative*** | 11 (37.9%) | 2 (50.0%) | 4 (25.0%) | 17 (34.7%) |
| ***QIAreach positive*** | 18 (62.1%) | 2 (50.0%) | 12 (75.0%) | 35 (65.3%) |

Supplementary Materials 7 - Sensitivity analysis reclassifying QIAreach QFT with time-to-positivity of 20 minutes as negative

1. QFT-Plus results according to QIAreach QFT result / level of positivity

|  | **QFT-Plus result** | | | **Total** |
| --- | --- | --- | --- | --- |
| **QIAreach result*** | **Negative** | **Positive** | **Indeterminate** |  |
| **Negative** | 588 (82%) | 59 (8%) | 70 (10%) | 717 |
| **Borderline positive, TTP 20 mins** | 133 (76%) | 23 (13%) | 19 (11%) | 175 |
| **Strong positive, TTP <20 mins** | 75 (48%) | 74 (47%) | 4 (4%) | 156 |

1. Test performance comparing QIAreach QFT (with positive results with time-to-positivity of 20 minutes reclassified to negative) with QFT-Plus

|  | **Overall** | **Age group (years)** | | |
| --- | --- | --- | --- | --- |
|  | **(n=1048*)** | **1 to 4**  **(n=674*)** | **10 to 19**  **(n=132)** | **20 to 40**  **(n=242)** |
| QIAreach result* |  |  |  |  |
| Negative | 717 (68%) | 505 (75%) | 91 (69%) | 121 (50%) |
| Borderline positive,  TTP 20 mins | 175 (17%) | 117 (17%) | 22 (17%) | 36 (15%) |
| Strong positive,  TTP <20 mins | 156 (15%) | 52 (8%) | 19 (14%) | 85 (35%) |
| Sensitivity of strong positive QIAreach (95% CI) ^1^ | 47%  (39%-56%) | 20%  (11%-31%) | 45%  (24%-68%) | 79%  (67%-89%) |
| Specificity of negative or borderline positive QIAreach (95% CI) ^1^ | 91%  (88%-93%) | 93%  (91%-95%) | 92%  (85%-97%) | 82%  (75%-87%) |
| Positive predictive value of strong positive QIAreach (95% CI) ^2^ | 50%  (41%-58%) | 28%  (16%-42%) | 56%  (31%-78%) | 62%  (50%-72%) |
| Negative predictive value of negative or borderline positive QIAreach (95% CI) ^2^ | 90%  (87%-92%) | 90%  (87%-92%) | 89%  (81%-94%) | 92%  (86%-95%) |
| Cohen’s kappa of reclassified QIAreach^2^ (95% CI) | 0.39  (0.32-0.45) | 0.15  (0.07-0.23) | 0.40  (0.23-0.58) | 0.56  (0.44-0.69) |

*Data on QIAreach QFT time-to-positivity missing for 1 participant ^1^Sensitivity and specificity of reclassified QIAreach QFT results calculated against comparator QFT-Plus positivity or negativity. “Strong positive” results defined as those with time to positivity less than 20 minutes. “Borderline positive” results, with a time to positivity of exactly 20 minutes, reclassified to negative. ^2^Indeterminate QFT-Plus values excluded for the purposes of calculating predictive values and Cohen’s kappa.

TTP: time-to-positivity. CI: Confidence interval. QIAreach: QIAreach QFT
